# Increased mortality in people with chronic multisite pain and insomnia: the HUNT study

**DOI:** 10.1016/j.sleepx.2025.100163

**Published:** 2025-11-17

**Authors:** Jonas Bloch Thorlund, Tom Ivar Lund Nilsen, Eivind Schjelderup Skarpsno

**Affiliations:** a Center for Muscle and Joint Health, Department of Sports Science and Clinical Biomechanics, University of Southern Denmark, Odense, Denmark; b Research Unit for General Practice, Department of Public Health, University of Southern Denmark, Odense, Denmark; c Department of Public Health and Nursing, Norwegian University of Science and Technology (NTNU), Trondheim, Norway; d Clinic of Emergency Medicine and Prehospital Care, St. Olavs Hospital, Trondheim, Norway

**Keywords:** Musculoskeletal pain, Insomnia, Sleep disturbances, Chronic pain, Widespread pain

## Abstract

**Objective:**

Insomnia is common among individuals with chronic musculoskeletal pain, and both conditions have been linked to mortality. However, the joint association of chronic multisite pain and insomnia with all-cause mortality is unknown, which we aimed to investigate in this study.

**Methods:**

We used data from 39,545 persons participating in the third wave of the Norwegian HUNT Study (2006–08) with complete information on both musculoskeletal pain and insomnia symptoms, linked to the national Cause of Death Registry. Hazard ratios (HRs) with 95 % confidence intervals (CIs) were calculated to assess the risk of death associated with the joint association of insomnia and chronic multisite pain.

**Results:**

Compared to individuals without chronic musculoskeletal pain and no insomnia, individuals with multisite chronic pain had a HR for all-cause mortality of 1.21 (95 % CI 1.01 to 1.43) if they reported insomnia and a HR of 1.00 (0.91–1.11) if they did not suffer from insomnia. Compared to the same reference category, pain-free individuals with insomnia had a HR of 1.05 (95 % 0.83 to 1.34). Our data showed no evidence of a synergistic effect between chronic multisite pain and insomnia on all-cause mortality.

**Conclusion:**

Individuals with chronic multisite pain and insomnia appeared to have higher all-cause mortality compared to pain-free persons without insomnia. Although speculative, these findings suggest that improving sleep quality in individuals with chronic multisite pain and insomnia may contribute to better overall health and potentially lower the risk of death from all causes.

## Highlights

• Multisite pain with insomnia was associated with 21 % increased mortality.
• No increased mortality related to pain or insomnia alone.
• No clear synergistic effect between multisite pain and insomnia on mortality.
• Improving sleep may reduce mortality in those with multisite pain and insomnia.

## 1 Introduction

Chronic musculoskeletal pain is a leading cause of disability and primary care use [1]. More than half of individuals with chronic musculoskeletal pain also report insomnia symptoms [2], making it a common, but often neglected comorbidity.

Chronic musculoskeletal pain has been associated with increased all-cause mortality and mortality specifically related to cancer and cardiovascular diseases, and this risk seems to be further amplified in individuals with multiple pain sites [3,4]. Similarly, increasing evidence suggest that insomnia is associated with increased risk of cancer and cardiovascular diseases [5,6], although findings have been conflicting. One explanation for the inconsistent findings is the use of different definitions of insomnia, where studies with stricter definitions of insomnia have shown stronger associations with all-cause mortality [7].

Chronic musculoskeletal pain and insomnia may exacerbate one another [8], and their co-occurrence may induce greater symptom severity and additional comorbidities [9]. Whether co-existence of these conditions also aggravates into a synergistic effect on overall mortality is not clear. We therefore aimed to prospectively investigate the joint association of chronic multisite musculoskeletal pain and insomnia with all-cause mortality in a population-based cohort.

## 2 Patients and methods

We used data from the third survey of the Trøndelag Health Study (The HUNT Study, 2006–08) [10]. All 93,860 inhabitants aged 20 years or more residing in the Nord-Trøndelag region were invited to participate and 50,807 (54 %) accepted the invitation. A detailed description of participation rates, questionnaires, and clinical examinations can be found at https://www.ntnu.edu/hunt. We included participants with complete information on both musculoskeletal pain and insomnia at the HUNT3 baseline survey.

### 2.1 Mortality outcome

We used the unique personal identification number of all Norwegian citizens to link each participant's record in the HUNT Study to information from the Norwegian Cause of Death Registry. Mortality data is based on death certificates reported by doctors, reporting the primary cause of death classified according to the International Classification of Disease (ICD). Both coverage and completeness for this registry is high, with medical information available for 98 % of all deaths [11]. Participants were followed up until death (all causes), emigration, or end of follow-up (December 31st, 2020).

### 2.2 Chronic musculoskeletal pain

Chronic musculoskeletal pain was assessed using the Standardised Nordic Musculoskeletal Questionnaire [12]. Presence of chronic musculoskeletal pain was assessed by the question: ‘During the last year, have you had pain and/or stiffness in muscles or joints that lasted for at least 3 consecutive months?’ with the response options “No” and “Yes”. Participants who reported to have chronic musculoskeletal pain were asked to indicate the affected body area(s), i.e., neck, shoulders, upper back, elbows, low back, hips, wrists/hands, knees, and ankles/feet. Participants were also asked the following question: “Have you been suffering from pain in both left and right sides of the body?”. Participants were defined to have chronic multisite pain if they reported chronic pain in the axial skeleton (i.e., neck, upper back, and/or low back), above (i.e., shoulders, elbows, wrists/hands) and below the waist (i.e., hip, knees, and/or ankles/feet, and in both left and right sides of the body.

### 2.3 Insomnia

Insomnia symptoms were assessed using four questions regarding the frequency (past three months), of difficulty falling asleep, frequent nighttime awakenings, early morning awakenings with inability to return to sleep, and experiencing daytime sleepiness. Participants were classified as having insomnia if they answered ‘several times a week’ on at least one of the nighttime symptoms and ‘several times a week’ on daytime sleepiness. This approximates the insomnia diagnosis according to the current international classification of sleep disorders (ICSD-3) [13]. Participants who answered ‘several times a week’ to at least one of the nighttime questions (i.e., difficulty falling asleep, frequent nighttime awakening, early morning awakening) but no daytime sleepiness, were classified as having sub-threshold insomnia.

### 2.4 Covariates

Body mass index (BMI) was calculated as weight in kilogrammes (kg) divided by the squared value of height in meters (m^2^). Leisure time physical activity was assessed using questions about the usual weekly frequency of exercise and the average duration and intensity of each session, and classified into ‘inactive”, ‘low’ (i.e. below recommended level), and ‘moderate/high’ (i.e. at or above the recommend level), according to recommendations for physical activity in adults at the time of the examination [14]. Smoking status was assessed by several questions related to current and past cigarette smoking and categorized as: ‘never smoked’, ‘former or occasional smoker’ and ‘current smoker’. Anxiety and/or depression were assessed using the Hospital anxiety and depression scale (HADS), which is a self-reported scale consisting of items on both depression and anxiety. Participants were considered as having anxiety and/or depression when surpassing the cut-off score in at least one of the scales [15].

### 2.5 Statistics

Cox regression was used to estimate hazard ratios (HR) of the joint association of chronic pain and insomnia with all-cause mortality. All analyses were adjusted for age (using age as the time scale in the model), sex, physical activity level (inactive, low, moderate/high), smoking (never, former or occasional, current smoker), and body mass index (continuous). Missing data on confounders were imputed using 20 imputed datasets, including all variables from for the main analyses and the sensitivity analyses. Relative excess risk due to interaction (RERI) was calculated to investigate the potential effect modification between the variables as departure from additive effect. Sensitivity analyses included (1) exclusion of the first 2 years of follow-up to avoid potential bias due to pre-existing disease at baseline (i.e. reverse causation); (2) exclusion of participants who reached 85 years of age during the follow-up to prevent age-related deflation of the estimates; (3) stratification of the follow-up period by 5 and 10 years to account for the possible underestimation of hazard ratios over long follow-ups; (4) adjustments for anxiety and/or depression due to the unclear temporal associations between multisite pain, insomnia, and mental health. All statistical analyses were performed using Stata for Windows, version 17 (StataCorp LP, College Station, TX, USA).

## 3 Results

In total, 39,545 (78 %) of 50,807 participants had complete data on both chronic musculoskeletal pain and insomnia symptoms. Of these, 52 % (n = 20,747) reported chronic musculoskeletal pain. Chronic multisite pain was reported by 17 % (n = 6876) of participants (Table 1).

**Table 1:** Baseline characteristics of study participants stratified by pain status (n = 39,545).

	No chronic pain	Chronic pain	Multisite pain
Participants, no. (%)	18,798	13,871	6876
Age, mean (SD)	51.8 (16.2)	55.6 (15.2)	57.9 (13.0)
Females, no. (%)	9630 (51)	7809 (56)	4779 (70)
Body mass index (kg/m^2^), mean (SD)∗	26.7 (4.2)	27.4 (4.4)	28.3 (4.8)
Insomnia, no. (%):			
No insomnia symptoms	14,598 (78)	8994 (65)	3171 (46)
Sub-threshold insomnia^a^	3474 (18)	3869 (28)	2628 (38)
Insomnia disorder^b^	726 (4)	1008 (7)	1077 (16)
Moderate-to-high physical activity level, no. (%)	8302 (44.2)	5436 (39.2)	2469 (35.9)
Current smoker, no. (%)	3823 (20.8)	3257 (24.2)	1873 (28.0)
History of cardiovascular disease, no. (%)	1777 (9.5)	1683 (12.1)	1085 (15.8)
History of cancer, no. (%)∗:	957 (5.1)	838 (6.0)	514 (7.5)
Anxiety and/or depression, no. (%)	1503 (8.0)	1840 (13.3)	1467 (21.3)

SD = Standard Deviation.

a At least one of the following symptoms: ‘difficulty falling asleep’, ‘difficulty maintaining sleep’, ‘waking up too early’, ‘daytime sleepiness’, but no daytime sleepiness.

b At least one nighttime insomnia symptom (‘difficulty falling asleep’, ‘difficulty maintaining sleep’, or ‘waking up too early’) accompanied by daytime sleepiness.

**Fig. 1:**
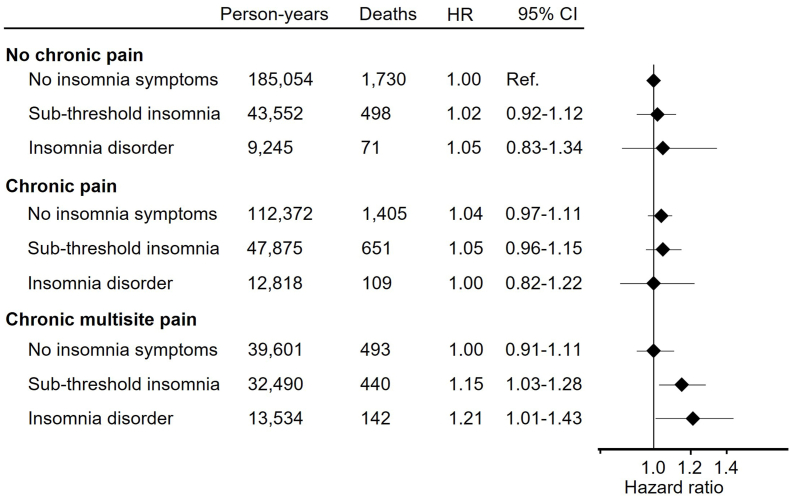
Association between chronic multisite pain combined with insomnia and all-cause mortality (n = 39,545). **Abbreviations**: CI = Confidence interval; HR = Hazard ratio. Sub-threshold insomnia was defined as the presence of at least one of the following symptoms: difficulty falling asleep, difficulty maintaining sleep, waking up too early, but no daytime sleepiness. Insomnia disorder was defined as at least one nighttime insomnia symptom (difficulty falling asleep, difficulty maintaining sleep, or waking up too early) accompanied by daytime sleepiness. Analyses were adjusted for age (years), sex (women, men), body mass index (kg/m^2^), occupation (classified according to the International Standard Classification of Occupations [ISCO]), smoking status (never, former or occasional, current), and physical activity level (inactive, low, moderate/high).

Compared to the reference group of individuals without chronic musculoskeletal pain and no insomnia symptoms, individuals with chronic multisite pain had a HR of 1.21 (95 % CI 1.01 to 1.43) if they reported insomnia disorder (Fig. 1). Compared to the same reference category, individuals with musculoskeletal pain had a HR of 1.00 (95 % CI 0.91–1.11) if they had no insomnia disorder and a HR of 1.15 (95 % CI 1.0 to 1.28) if they reported sub-threshold insomnia, whereas individuals without chronic pain had a HR of 1.05 (95 % CI 0.83 to 1.34) if they had insomnia disorder (Fig. 1). Despite the increased mortality among those with chronic multisite pain and insomnia, there was no evidence of a synergistic effect beyond additivity for the co-existence of these conditions (RERI = 0.16 [95 % CI -0.18 to 0.55]). Sensitivity analyses had negligible influence on the results (data not shown).

## 4 Discussion

This population-based study showed that individuals reporting insomnia alongside chronic multisite pain had a 21 % higher risk of all-cause mortality than individuals without chronic pain and no insomnia symptoms. However, we observed no evidence of a synergistic effect of chronic multisite pain and insomnia on mortality. These results suggest that addressing insomnia symptoms should be part of the management strategy for these individuals.

This study has some limitations. Reverse causation from pre-existing health conditions (e.g., cardiovascular disease or cancer) cannot be ruled out, though sensitivity analyses excluding deaths within the first 2 years of follow-up or affected individuals showed similar results. More detailed information about various sleep characteristics is warranted to fully understand the potentially interplay between sleep and pain on mortality. Including participants at all ages may affect mortality risk but analyses excluding participants under 45 or censoring at 85 years gave largely similar results as the main analyses. Due to few events, especially among those with insomnia, we could not analyze cause-specific mortality or mortality in subgroups. Furthermore, it should be noted that the questions used to assess insomnia have varying degrees of reliability [16].

Previous research has suggested that chronic musculoskeletal pain and insomnia symptoms are independently associated with increased mortality, mainly from cancer and cardiovascular diseases, and that mortality is further increased in those with multiple pain sites and more severe insomnia [3,4,7]. This is the first study to investigate the joint association of insomnia and chronic musculoskeletal pain on mortality. While no synergistic effect of chronic multisite pain and insomnia severity was observed, having both chronic multisite pain and comorbid insomnia was associated with a 21 % higher all-cause mortality compared to those without chronic pain and no insomnia. Notably, we found no evidence of increased mortality among individuals with insomnia alone or chronic pain alone. The exact mechanism underlying these findings remains uncertain but may involve underlying undiagnosed diseases or factors such as greater physical disability and elevated emotional stress among those with co-occurring multisite pain and insomnia disorder [9,17]. Thus, our findings indicate that individuals experiencing insomnia disorder alongside multisite chronic pain and insomnia may benefit from individualized sleep therapy as part of their treatment, potentially contributing to a reduction in overall mortality.

## CRediT authorship contribution statement

**Jonas Bloch Thorlund:** Writing – review & editing, Writing – original draft, Visualization, Methodology, Investigation, Formal analysis, Conceptualization. **Tom Ivar Lund Nilsen:** Writing – review & editing, Visualization, Project administration, Methodology, Investigation, Formal analysis, Conceptualization. **Eivind Schjelderup Skarpsno:** Writing – review & editing, Writing – original draft, Visualization, Project administration, Methodology, Investigation, Formal analysis, Conceptualization.

## Data sharing

This study utilized data from the HUNT Study (https://www.ntnu.edu/hunt). Research groups with a Principal Investigator affiliated with a Norwegian research institute can apply for access to this data. Researchers from non-Norwegian countries must collaborate with a Norwegian partner to use data from the HUNT Study. Each project requires approval from the HUNT Data Access Committee, the Regional Medical Ethical Committee, and, in some cases, the Data Inspectorate. Due to participant confidentiality, the data are not publicly available.

## Funding

No funding to report for this study.

## Declaration of competing interest

The authors declare the following financial interests/personal relationships which may be considered as potential competing interests: Jonas Bloch Thorlund reports a relationship with Novo Nordisk Foundation that includes: funding grants. The other authors declare that they have no known competing financial interests or personal relationships that could have appeared to influence the work reported in this paper.
